# Draft Genome Sequence of the Plant Growth-Promoting Sphingobium sp. Strain AEW4, Isolated from the Rhizosphere of the Beachgrass Ammophila breviligulata

**DOI:** 10.1128/genomeA.00410-18

**Published:** 2018-05-24

**Authors:** Abanoub E. Wanees, Shari J. Zaslow, Savannah J. Potter, Brandon P. Hsieh, Brianna L. Boss, Javier A. Izquierdo

**Affiliations:** a Department of Biology, Hofstra University, Hempstead, New York, USA

## Abstract

Sphingobium sp. strain AEW4 is a novel isolate from rhizosphere soil attached to the root of the American beachgrass Ammophila breviligulata. The genomic sequence consisted of 4,678,518 bp and 4,428 protein-coding sequences. Here we report the draft genome sequence of this strain and some initial insights on its plant growth-promoting capabilities.

## GENOME ANNOUNCEMENT

Rhizospheric plant growth-promoting bacteria (PGPB) are able to stimulate increases in yield, reduce the infective effect of pathogens, and increase plant survival in the face of biotic or abiotic plant stressors (1). Sphingobium sp. strain AEW4 was isolated from rhizosphere soil attached to the root of the American beachgrass Ammophila breviligulata at Cedar Beach, Jones Beach Island, New York, in the United States (40°37′N, 73°21′W). Based on its 16S rRNA gene sequence, its closest match was Sphingobium xenophagum, an organism able to degrade xenobiotic aromatic compounds (2, 3). Initial characterization of the plant growth-promoting properties of this organism revealed that it is able to produce siderophores and indole-3-acetic acid, as well as to induce root growth. There are various examples of plant growth-promoting organisms within the Sphingomonadaceae (4–6), but the genus Sphingobium is largely limited to organisms that are able to break down xenobiotic compounds (7).

Genomic DNA of Sphingobium sp. strain AEW4 was obtained using the GenElute genomic DNA isolation kit (Sigma-Aldrich). An Illumina library was prepared using a Nextera DNA sample preparation kit (Illumina) according to the manufacturer's user guide. The initial concentration of DNA was determined to be 28.2 ng/µl using the Qubit double-stranded DNA (dsDNA) HS assay kit (Life Technologies). A total of 50 ng of DNA was used to prepare the library. The sample underwent simultaneous fragmentation and addition of adapter sequences during a limited-cycle (5 cycles) PCR in which unique indices were added to the sample. Following the library preparation, the final concentration of the library (15.1 ng/µl) was measured using the Qubit dsDNA HS assay kit (Life Technologies), and an average library size of 776 bp was determined using the Agilent 2100 Bioanalyzer (Agilent Technologies). The library was then diluted to 10 pM and clustered using the cBot (Illumina) and paired end sequenced for 500 cycles using the HiSeq 2500 system (Illumina). Assemblies were created using SeqMan NGen from the Lasergene genomics package version 12.1.0 (DNAStar, Madison, WI). Annotation was conducted with the NCBI Prokaryotic Genome Annotation Pipeline (8) and with Rapid Annotations using Subsystems Technology (RAST) server, version 2.0 (9). The antiSMASH platform (10) was used for the identification of biosynthetic gene clusters.

The final assembly of the genome of Sphingobium sp. strain AEW4 consisted of 4,678,518 bp with a G+C content of 62.6% and 4,428 protein-coding sequences. Initial findings reveal distinct differences from the genomes of closely related Sphingobium xenophagum strains, NBRC 107872 and QYY (11). In particular, the genome Sphingobium sp. AEW4 contains unique genes involved in the synthesis of siderophores, the utilization of various monosaccharides, and genes that are essential for acetoin, butanediol, and butyrate fermentation. These are all promising key processes that may confer this organism with plant growth-promoting properties in interactions with the beachgrass *Ammophila breviligulata*, which will be the focus of future studies.

### Accession number(s).

The genome sequence of Sphingobium sp. strain AEW4 has been deposited in GenBank under the accession no. PYGL00000000.
